# Quill Mites of the Family Syringophilidae (Acariformes: Cheyletoidea) Parasitising Birds of the Subfamily Euphoninae (Passeriformes: Fringillidae)

**DOI:** 10.3390/ani15050764

**Published:** 2025-03-06

**Authors:** Bozena Sikora, Markus Unsoeld, Roland R. Melzer, Stefan Friedrich, Martin Hromada, Maciej Skoracki

**Affiliations:** 1 Department of Animal Morphology, Faculty of Biology, Adam Mickiewicz University, 61-614 Poznań, Poland; 2 SNSB-Bavarian State Collection for Zoology, Ornithology Section, 81247 Munich, Germany; unsoeld@snsb.de; 3 SNSB-Bavarian State Collection for Zoology, Arthropoda varia Section, 81247 Munich, Germany; melzer@snsb.de (R.R.M.); friedrich@snsb.de (S.F.); 4 Faculty of Biology, Ludwig Maximilian University of Munich, 82152 Planegg-Martinsried, Germany; 5 GeoBio-Center, Ludwig Maximilian University of Munich, 80333 Munich, Germany; 6 Laboratory and Museum of Evolutionary Ecology, Department of Ecology, Faculty of Humanities and Natural Sciences, University of Presov, 080 01 Presov, Slovakia; hromada.martin@gmail.com

**Keywords:** acari, aves, biodiversity, birds, ectoparasites, euphoninae, fringillidae, syringophilidae

## Abstract

Quill mites of the family Syringophilidae (Acariformes: Prostigmata) are highly specialised avian ectoparasites that inhabit feather quills. Despite their widespread occurrence, their diversity, distribution, and host associations remain poorly understood. This study examined the diversity and ecological interactions of syringophilid mites parasitising Euphoninae hosts. We analysed 298 dry bird skins representing 25 species deposited in the Bavarian State Collection for Zoology in Munich, Germany. Quill mite infestations were detected in 15 host species, identifying 4 mite species, including 2 newly described taxa: *Aulonastus neotropicalis* sp. n. and *Syringophilopsis euphonicus* sp. n. Infestation prevalence ranged from 2% to 25%. Quill mite–host interactions exhibited high specialisation and niche differentiation, with no co-occurring species sharing the same microhabitat. Network analysis indicated moderate connectance (0.35) and significant host specificity (H2′ = 0.77, *p* = 0.007). Biogeographic history suggests that divergence from Carduelinae and subsequent evolutionary events shaped syringophilid diversity in Euphoninae. These findings underscore the importance of museum collections in uncovering cryptic parasite diversity and provide new insights into host–parasite co-evolutionary dynamics.

## Simple Summary

Birds and their parasites have evolved over millions of years, forming complex relationships that shape biodiversity. Until now, little was known about the mites living inside the feathers of Euphoninae birds, which includes *Euphonia* and *Chlorophonia* species found in Central and South America. Previously, only one species of these mites was recorded in this bird group. In our study, we discovered 4 species of quill mites living in the feathers of 15 bird species, significantly expanding our knowledge of their diversity and host associations. Our findings suggest that the evolutionary history of these birds has influenced which mite species they host. Some mites are found in both *Euphonia* and *Chlorophonia*, while others appear restricted to only one of these genera. Additionally, our findings suggest that different mite species occupying the same ecological niche tend to avoid co-infesting the same bird species, likely as a strategy to minimise competition. The mite material collected from museum bird collections provides valuable insights into host–parasite relationships, helping scientists better understand biodiversity, evolution, and species interactions in nature.

## 1. Introduction

The subfamily Euphoninae, belonging to the family Fringillidae (order Passeriformes), represents a group of birds native to the tropical and subtropical regions of Central and South America [1,2,3]. This subfamily comprises two genera, *Euphonia* and *Chlorophonia*, encompassing 35 species characterised by their striking plumage and specialised dietary habits [1,4]. Members of Euphoninae are predominantly frugivorous, although occasional insect consumption has been reported. Their role as seed dispersers is ecologically significant, contributing to the maintenance and regeneration of tropical forests. Morphologically, species within *Euphonia* and *Chlorophonia* exhibit sexual dimorphism, with males often adorned in vibrant combinations of yellow, blue, and green, while females display more subdued hues that aid in camouflage. These birds inhabit various forested environments, from lowland rainforests to montane cloud forests, where they form small social groups or pairs [1].

Quill mites of the family Syringophilidae (Acariformes: Prostigmata: Cheyletoidea) are highly specialised avian parasites inhabiting feather quills [5]. Quill mites demonstrate high specificity toward their hosts, with most syringophilid species being either monoxenous or oligoxenous parasites. They also show distinct preferences for the specific habitats they colonise [5,6]. The Syringophilidae family includes approximately 400 species across 63 genera, associated with 27 bird orders [7,8]. Until recently, new species of the family Syringophilidae were described rather randomly, without much reference to host groups. It is only in recent years that intensive studies on quill mite fauna have begun, focusing on entire bird families or orders [9,10,11,12,13].

Up to now, the acarofauna of mites from the family Syringophilidae parasitising Euphoninae birds comprised only a single record—*Syringophilopsis stawarczyki* Skoracki et al., 2010 described from *Chlorophonia cyanocephala* in Brazil [14]. This limited record highlights the significant knowledge gap regarding the diversity, distribution, and host-parasite interactions of Syringophilidae mites in Euphoninae. This study addresses this gap by expanding the knowledge of Syringophilidae mites associated with Euphoninae and exploring their potential implications for host and parasite ecology.

## 2. Materials and Methods

### 2.1. Mites Collection, Preparation, Description, and Deposition

Mite material used in this study was collected from dry bird skins housed in the Bavarian State Collection for Zoology (SNSB-ZSM) in Munich, Germany. For each bird specimen, approximately ten contour feathers near the cloacal region, two under-tail coverts, two upper-tail coverts, and one secondary wing covert were selected. These feathers were subsequently examined for the presence of quill mites belonging to the family Syringophilidae.

Infested quills were placed in Nesbitt’s solution for three days at room temperature to soften the mites inside. Each quill was then carefully opened along its length using fine-tipped forceps. The mites were rinsed in 70% ethanol and subsequently mounted on permanent microscope slides using Hoyer’s medium [15]. Slide-mounted mites were examined using a ZEISS Axioscope light microscope (Carl Zeiss AG, Oberkochen, Germany) equipped with differential interference contrast (DIC) optics and a camera lucida.

Descriptions of idiosomal setation follow the system established by Grandjean [16], as adapted for Prostigmata by Kethley [17]. The nomenclature for leg chaetotaxy adheres to Grandjean’s proposal [18], while the morphological terminology is based on the works of Kethley [5] and Skoracki [6]. All measurements are reported in micrometres, with ranges for paratypes provided in brackets following the holotype data.

All mite specimens analysed in this study are deposited in two institutions: Adam Mickiewicz University, Poznan, Department of Animal Morphology (AMU), and the Bavarian State Collection for Zoology, Munich, Germany (SNSB-ZSM).

### 2.2. Statistics

Descriptive statistics were calculated using Quantitative Parasitology v.3.0 on the Web [19,20,21]. To analyse the host–parasite ecological two-way web, we employed the “bipartite” package in R version 4.3.1 [22,23], quantifying the ecological relationships between parasites and their hosts. In this analysis, parasite prevalence served as a quantitative index. First, we calculated connectance, a measure of the ratio of actual connections in the bipartite network to the maximum possible connections. Next, we assessed the C-score, a metric that quantifies the tendency of species pairs to avoid co-occurrence. A high C-score suggests that species rarely share the same host, which may indicate competition or niche differentiation, whereas a low value implies frequent co-occurrence, suggesting random or overlapping associations. To evaluate whether parasites were generalists or specialists, we used the H2′ metric, a network-wide specialisation index. H2′ ranges from 0 (complete generalisation, where all species interact with many partners) to 1 (complete specialisation, where each species interacts with only a single partner or a very restricted set). This metric provides a standardised way to compare specialisation across different networks. The null.*t*.test was applied to determine whether the observed H2′ values significantly deviated from random expectations [23], helping to assess whether the observed level of specialisation is ecologically meaningful. In the next step, we calculated nestedness, which describes the degree of order in the network, where parasites with fewer host interactions tend to share hosts with parasites with more interactions. Finally, to measure the functional diversity of parasites, we calculated d′, which represents how unique a species interaction pattern is relative to the entire network. The metric d′ is normalised between 0 and 1, where 0 indicates that a species interacts similarly to other species in the network (low functional diversity), while 1 represents a highly unique interaction pattern, suggesting a high degree of specialisation.

### 2.3. Bird Systematics and Zoogeographical Regions

The scientific names and taxonomy of birds are based on Winkler et al. [1] and Clements et al. [24]. Host species distribution follows BirdLife International [25]. Zoogeographical regions are defined according to Holt et al. [26] and Ficetola et al. [27].

## 3. Results

### 3.1. Systematics

Family Syringophilidae Lavoipierre, 1953

Subfamily Syringophilinae Lavoipierre, 1953

#### 3.1.1. *Aulonastus neotropicalis* sp. n. (Figure 1 and Figure 2)

Female, holotype. Total body length 465 (460–475 in nine paratypes). Gnathosoma. Infracapitulum apunctate. Each medial branch of peritremes has two chambers; each lateral branch has five chambers. Stylophore 130 (130–140) long; exposed portion of stylophore apunctate, 100 (100–105) long. Idiosoma. Propodonotal shield rectangular in shape, apunctate, bearing bases of setae *ve*, *si*, *c1*, and *se*. Length ratio of setae *ve*:*si* 1:1. Setae *se* and *c1* situated at same transverse level. Setae *c1* 1.2 times longer than *se*. Length ratio of setae *d2*:*c1* 1:1.2. Hysteronotal shield fused to pygidial shield, weakly sclerotised, apunctate, bases of setae *d1* and *e2* situated near this shield. Setae *f2* 3–3.5 times longer than *f1*. Setae *h2* 4–4.4 times longer than *f2*. Agenital setae *ag1* 1.3–1.7 longer than *ag2*. Genital plate absent. Both pairs of genital setae subequal in length. All coxal fields apunctate. Setae *3c* 2.8 times longer than *3b*. Legs. Fan-like setae *p’* and *p″* of legs III and IV with five or six tines. Lengths of setae: *ve* 15 (15–20), *si* 20 (15–20), *se* 160 (160–180), *c1* 190 (190–210), *c2* 155 (150–165), *d1* 20 (15–20), *d2* 165 (130–165), *e2* 15 (15–20), *f1* 20 (20–25), *f2* 70 (70–80), *h1* 20 (20–25), *h2* 310 (270–320), *ps1* 20 (15–20), *g1* and *g2* 20 (20–25), *ag1* 65 (65–85), *ag2* 50 (40–50), *ag3* 105 (105–110).

Male. Total body length 260–270 in two paratypes. Gnathosoma. Infracapitulum apunctate. Each medial branch of peritremes has two chambers; each lateral branch has five or six chambers. Stylophore 100–105 long; exposed portion of stylophore apunctate, 80 long. Idiosoma. Propodonotal shield invisible in posterior part, apunctate, bearing bases of setae *ve*, *si*, and *c1*, bases of setae *se* on or near this shield. Length ratio of setae *ve*:*si* 1:1. Bases of setae *se* situated anterior to level of setae *c1*. Setae *c1* and *se* subequal in length. Setae *d2* short but twice as long as *d1* and *e2*. Hysteronotal and pygidial shields absent. Setae *h2* 3–4 times longer than *f2*. Two pairs of agenital setae present, *ag1* 2–2.5 times longer than *ag2*. All coxal fields apunctate. Lengths of setae: *ve* 5–10, *si* 5–10, *se* 20, *c1* 20, *c2* 10–15, *d1* 10, *d2* 5, *e2* 5, *f2* 10, *h2* 30–40, *ag1* 20–25, *ag2* 10.

**Figure 1:**
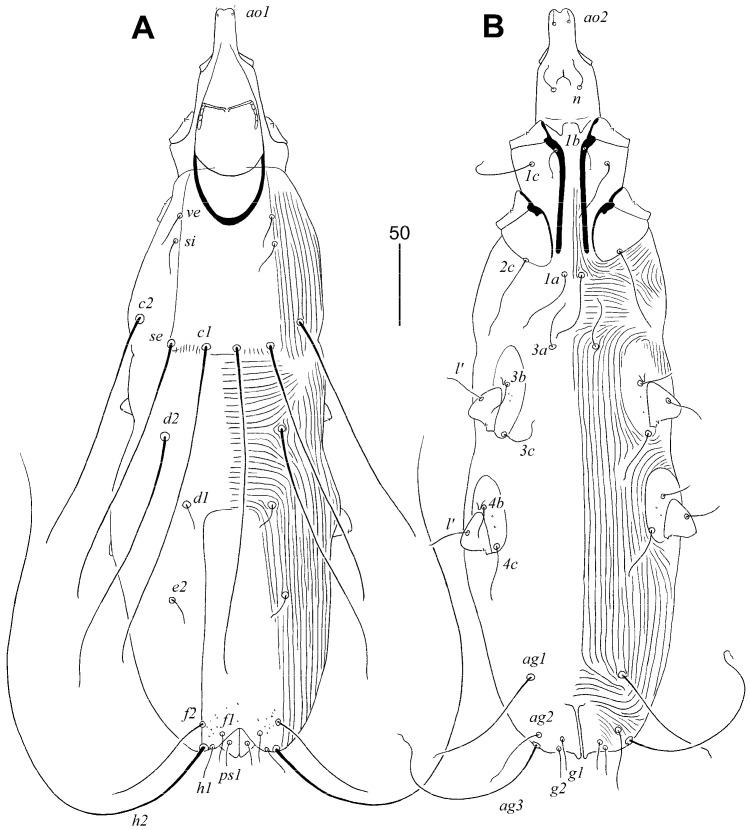
*Aulonastus neotropicalis* sp. n., female. (**A**) Dorsal view; (**B**) ventral view.

**Figure 2:**
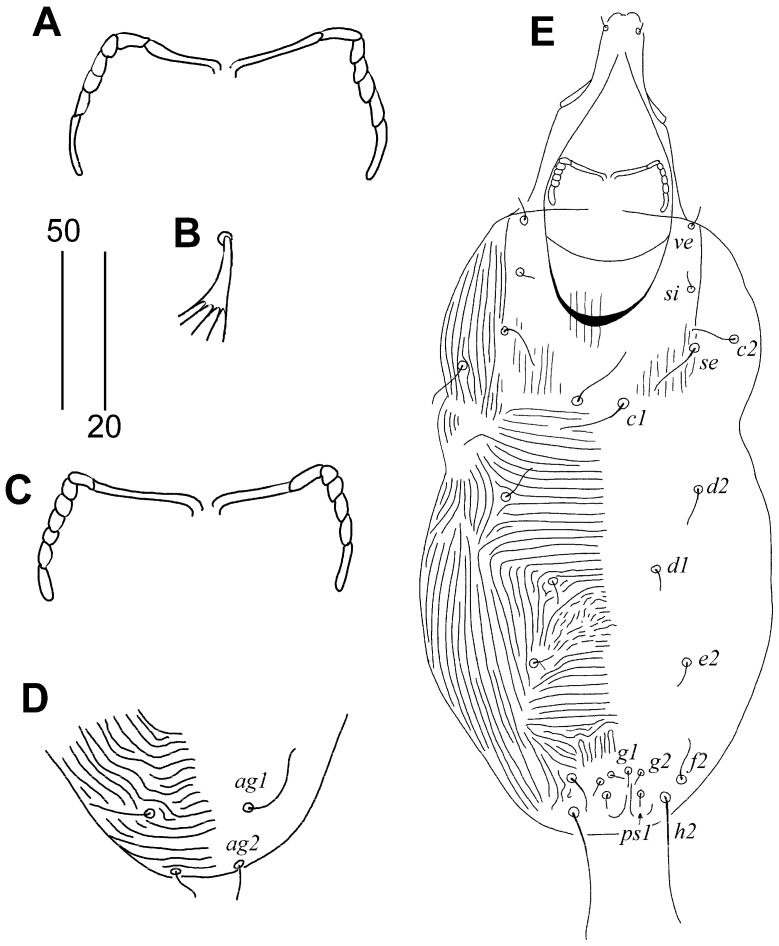
*Aulonastus neotropicalis* sp. n., female (**A**,**B**): (**A**) Peritremes; (**B**) fan-like seta *p’III*. Male (**C**,**D**): (**C**) Peritremes; (**D**) opisthosoma in ventral view. (**E**) Body in dorsal view. Scale bars (**A**–**C**) = 20 µm, (**D**,**E**) = 50 µm.

##### Type Material

Female holotype, nine female paratypes, and two male paratypes from the Blue-naped Chlorophonia *Chlorophonia cyanea* (Thunberg) (host reg. no. SNSB-ZSM 30.96); Venezuela: Carabobo State, Valencia, 1928, coll. P. C. Vogl.

##### Type Material Deposition

Holotype and most paratypes deposited in the SNSB-ZSM (reg. no. ZSMA20250001) except two female paratypes and one male paratype in the AMU (reg. no. AMU-MS-24-1025-012).

##### Additional Material

Ex type host species (host reg. no. SNSB-ZSM 27.1872); Bolivia: Santa Cruz Province, Buena Vista, August 1926, coll. M. Kiefer—three females and two males deposited in the SNSB-ZSM (reg. no. ZSMA20250002), one female and one male in the AMU (reg. no. AMU-MS-24-1025-010). From the same host species (host reg. no. SNSB-ZSM 10.2153); Venezuela: Carabobo State, Valencia, 9 February 1910, coll. S. M. Klages—two females deposited in the AMU (reg. no. AMU-MS-24-1025-013). From the same host species (host reg. no. SNSB-ZSM 09.2281); Venezuela: Sucre State, Bermúdez, March 1897, coll. E. Andre—one female deposited in the AMU (reg. no. AMU-MS-24-1025-014).

Ex the Lesser Antillean Euphonia *Chlorophonia flavifrons* (Sparrman) (host reg. no. SNSB-ZSM 09/2291); Guadeloupe (Lesser Antillean Creole): 16 January 1896, coll. Chazalie—three females deposited in the SNSB-ZSM (reg. no. ZSMA20250003), one female and one male in the AMU (reg. no. AMU-MS-24-1025-034).

Ex the Golden-rumped Euphonia *Chlorophonia cyanocephala* (Vieillot) (host reg. no. SNSB-ZSM 09/2314); Colombia: Bogota, 1896, no other data—three females deposited in the SNSB-ZSM (reg. no. ZSMA20250004), one female in the AMU (reg. no. AMU-MS-24-1025-037).

Ex the Scrub Euphonia *Euphonia affinis* (Lesson) (host reg. no. SNSB-ZSM 15.941); Colombia: Bogotá D.C., Bogota, no other data—three females deposited in the SNSB-ZSM (reg. no. ZSMA20250005), one female and one male in the AMU (reg. no. AMU-MS-24-1025-016). From the same host species (host reg. no. SNSB-ZSM 09.5215) and locality—two females deposited in the AMU (reg. no. AMU-MS-24-1025-015).

Ex the Velvet-fronted Euphonia *Euphonia concinna* Sclater (host reg. no. SNSB-ZSM 09/2356); Colombia: no other data—one female deposited in the SNSB-ZSM (reg. no. ZSMA20250006), one female in the AMU (reg. no. AMU-MS-24-1025-023). From the same host species (host reg. no. SNSB-ZSM 03.767); Colombia: Cauca Valley, 1903, coll. J.H. Batty—one female deposited in the AMU (reg. no. AMU-MS-24-1025-024).

Ex the Yellow-crowned Euphonia *Euphonia luteicapilla* (Cabanis) (host reg. no. SNSB-ZSM 09.5337); Panama: Chiriquí Province, 17 February 1905, coll. H. Watson—three females and one male deposited in the SNSB-ZSM (reg. no. ZSMA20250007), two females and one male in the AMU (reg. no. AMU-MS-24-1025-025).

Ex the Tawny-capped Euphonia *Euphonia anneae* Cassin (host reg. no. SNSB-ZSM 09.2303); Costa Rica: Guanacaste Province, Puerto Carrillo, 21 November 1897, coll. C.F. Underwood—three females and two males deposited in the SNSB-ZSM (reg. no. ZSMA20250008), one female and one male in the AMU (reg. no. AMU-MS-24-1025-043).

Ex the Orange-bellied Euphonia *Euphonia xanthogaster* Sundevall (host reg. no. SNSB-ZSM 16.304); Peru: Carabaya Andes, Puno Region, Chaquimayo, 2 June 1910, coll. Watkins—four females deposited in the SNSB-ZSM (reg. no. ZSMA20250009), two females in the AMU (reg. no. AMU-MS-24-1025-047). From the same host species (host reg. no. SNSB-ZSM 16.303) and locality, 18 June 1910, coll. Watkins—two females in the AMU (reg. no. AMU-MS-24-1025-048).

Ex the White-lored Euphonia *Euphonia chrysopasta* Sclater & Salvin (host reg. no. SNSB-ZSM 09.2364); Venezuela: Río Caura, coll. E. Andre—three females and one male deposited in the SNSB-ZSM (reg. no. ZSMA20250010), two females and one male in the AMU (reg. no. AMU-MS-24-1025-041).

Ex the Golden-sided Euphonia *Euphonia cayennensis* (Gmelin) (host reg. no. SNSB-ZSM 1909/275); French Guiana: 1907, coll. Le Moult—three females and one male deposited in the SNSB-ZSM (reg. no. ZSMA20250011), three females and one male in the AMU (reg. no. AMU-MS-24-1025-050).

##### Differential Diagnosis

*Aulonastus neotropicalis* sp. n. is morphologically similar to *A. fringillus* Skoracki, 2011 recorded from the Common Chaffinch *Fringilla coelebs* Linnaeus and the Eurasian Linnet *Linaria cannabina* (Linnaeus) [6,28]. In females of both species, the propodonotal shield is rectangular and bears bases of setae *ve*, *si*, *se*, and *c1*; the hysteronotal shield is fused with the pygidial shield; setae *c1* are 1.2–1.5 longer than *se*; and setae *e2* and *d1* are subequal in length. This new species differs from *A. fringillus* as follows: in females of *A. neotropicalis* sp. n., the propodonotal shield is apunctate; the coxal fields III and IV are sparsely punctate; the lengths of setae *se*, *c1*, *c2*, and *d2* are 160–180, 190–210, 150–165, and 130–165 respectively. In females of *A. fringillus*, the propodonotal shield is punctate on the whole surface; the coxal fields III and IV are apunctate; the lengths of setae *se*, *c1*, *c2*, and *d2* are 120–125, c1 140–165, c2 90–100, and 80–90 respectively.

##### Etymology

The species name “*neotropicalis*” reflects the Neotropical origin of its hosts.

#### 3.1.2. *Syringophilopsis euphonicus* sp. n. (Figure 3, Figure 4 and Figure 5)

Female, holotype. Total body length 980 (950–1040 in three paratypes). Gnathosoma. Hypostomal apex with one pair of small and sharp-ended protuberances. Infracapitulum apunctate. Stylophore 250 (230–250) long; exposed portion of stylophore apunctate, 205 (200–205) long. Length of movable cheliceral digit 145 (145–150). Each medial branch of peritremes has 2 or 3 chambers, each lateral branch with 12 chambers. Idiosoma. Propodonotal shield concave on anterior margin, sculptured laterally, and sparsely punctate near bases of setae *ve* and *si*, bearing bases of setae *vi*, *ve*, *si*, and *c1*; bases of *se* situated on the margin of this shield. Bases of setae *se* and *c1* situated at same transverse level. Bases of setae *c2* situated posterior to level of *si* setal bases. Length ratio of setae *vi*:*ve*:*si* 1:1.5–2:3.5–4. Hysteronotal shields absent. Pygidial shield weakly sclerotised in anterior part, apunctate. Genital setae *ag2* 3.8–4 times longer than genital setae *g1* and *g2*. Setae *ag1* and *ag2* long and subequal in length. Coxal fields I–II sparsely punctate, III–IV densely punctate. Apodemes I fused to apodemes II in anterior part of apodemes II. Coxal setae *3c* twice as longer as *3b*. Lengths of setae: *vi* 90 (90–95), *ve* 140 (150–180), *si* 315 (350–355), *se* 335 (370–390), *c1* 320 (375–380), *c2* 325 (350), *d1* 320 (370–410), *d2* 320 (360), *e2* 300 (375–400), *f1* longer than 250, *f2* 400, *h1* 290 (315–345), *h2* 430 (415–470), *ps1* and *ps2* 30 (25–35), *g1* and *g2* 50 (35–45), *ag1* 195 (230–295), *ag2* 190 (230–235), *ag3* 230 (230–300), *l’RIV* 60 (40–60), *tc’III–IV* 65 (65–70), *3b* 80 (100–110), *3c* 160 (150).

**Figure 3:**
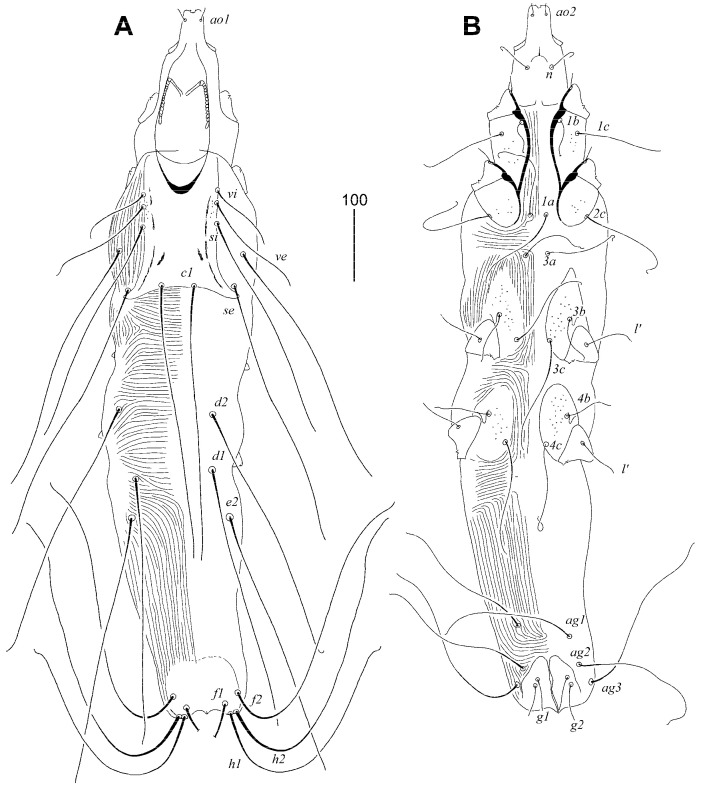
*Syringophilopsis euphonicus* sp. n., female. (**A**) Dorsal view; (**B**) ventral view.

**Figure 4:**
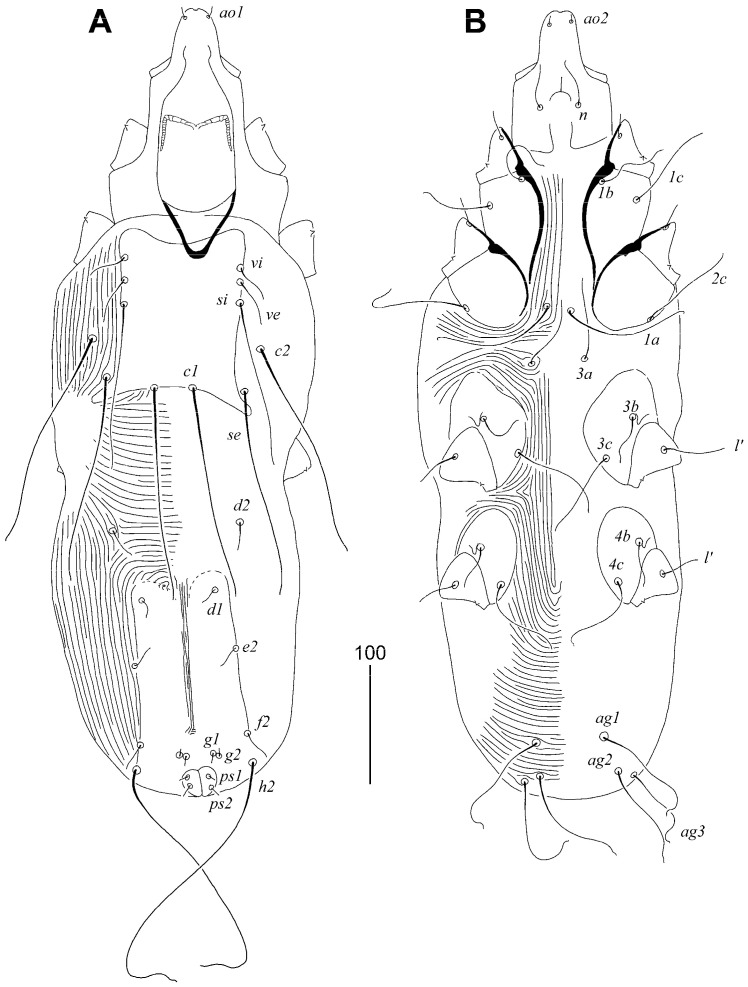
*Syringophilopsis euphonicus* sp. n., male. (**A**) Dorsal view; (**B**) ventral view.

**Figure 5:**
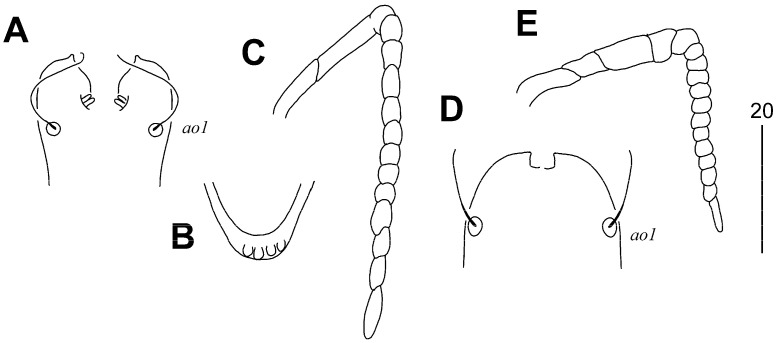
*Syringophilopsis euphonicus* sp. n., female (**A**–**C**): (**A**) Hypostomal apex; (**B**) posterior part of stylophore; (**C**) peritreme. Male (**D**,**E**): (**D**) Hypostomal apex; (**E**) peritreme.

Male, paratype. Total body length 670. Gnathosoma. Hypostomal apex without protuberances. Infracapitulum apunctate. Stylophore 200 long; exposed portion of stylophore apunctate, 160 long. Each medial branch of peritremes with 5–6 chambers, each lateral branch with 12 chambers. Idiosoma. Propodonotal shield rectangular in shape, concave on anterior margin, apunctate, bearing bases of setae *vi*, *ve*, *si*, and *c1*; bases of setae *se* situated on the margin of this shield. Bases of setae *se* and *c1* situated at same transverse level. Bases of setae *c2* situated posterior to level of *si* setal bases. Hysteronotal shield fused to pygidial shield, divided longitudinally, apunctate, bearing bases of setae *d1*, bases of setae *e2*, *f2*, and *h2* situated on the margin of this shield. Three pairs of agenital setae present, all subequal in length. Coxal fields I–IV apunctate. Apodemes I fused to apodemes II in middle part of apodemes II. Lengths of setae: *vi* 30, *ve* 40, *si* 140, *se* 170, *c1* 170, *c2* 175, *d1* 20, *d2* 30, *e2* 20, *f2* 35, *h2* 245, *ag1* 90, *ag2* 95, *ag3* 100, *l’RIII* 50, *3b* 60, *3c* 85.

##### Type Material

Female holotype, three female paratypes and one male paratype from the Trinidad Euphonia *Euphonia trinitatis* Strickland (host reg. no. SNSB-ZSM 09.2381); Venezuela: Bermúdez, Andes of Cumaná, March 1897, coll. E. Andre.

##### Type Material Deposition

Holotype and two female paratypes are deposited in the SNSB-ZSM (reg. no. ZSMA20250012), one female paratype and one male paratype in the AMU (reg. no. MS-24-1025-018B).

##### Additional Material

Ex the Orange-bellied Euphonia *Euphonia xanthogaster* Sundevall (host reg. no. SNSB-ZSM 16.304); Peru: Carabaya Andes, Puno Region, Chaquimayo, 2 June 1910, coll. Watkins—two females deposited in the SNSB-ZSM (reg. no. ZSMA20250013), two females and one male in the AMU (reg. no. MS-24-1025-047). From the same host species (host reg. no. SNSB-ZSM 16.303) and locality, 18 June 1910, coll. Watkins—one female deposited in the AMU (reg. no. AMU-MS-24-1025-048B). From the same host species (host reg. no. SNSB-ZSM 12.587); Peru: Puno Region, Carabaya Province, Yahuarmayo, 4 December 1910, coll. Watkins—one female deposited in the AMU (reg. no. MS-24-1025-046). From the same host species (host reg. no. SNSB-ZSM 12.588); Peru: Puno Region, Carabaya Province, Yahuarmayo, 4 December 1910, coll. Watkins—two females deposited in the AMU (reg. no. MS-24-1025-049).

Ex the White-vented Euphonia *Euphonia minuta* Cabanis (host reg. no. SNSB-ZSM 67.122); Colombia: Antioquia Department, Mutatá, 120 m a.s.l., 13 August 1966, coll. J. Haffer—two females deposited in the SNSB-ZSM (reg. no. ZSMA20250014), one female in the AMU (reg. no. MS-24-1025-045). From the same host species (host reg. no. SNSB-ZSM 09.2352); Colombia: 1896, no other data—one female deposited in the AMU (reg. no. MS-24-1025-044).

##### Differential Diagnosis

*Syringophilopsis euphonicus* sp. n. belongs to the *elongatus* species-group [6]. Within this group, it is morphologically similar to *S. nucifragus* Skoracki, 2011, which has been recorded from the Spotted Nutcracker *Nucifraga caryocatactes* (Linnaeus) (Passeriformes: Corvidae) from Europe [6]. In females of both species, setae *vi* are shorter than 160 µm, the hysteronotal shields are absent, and agenital setae *ag2* are distinctly longer (3–4 times) than genital setae (*g1* and *g2*). The new species, *S. euphonicus* sp. n., can be distinguished by the following features: in females of *S. euphonicus* sp. n., the hypostomal apex is ornamented with a single pair of small and sharp-ended protuberances; the length of setae *vi* is 90–95 µm; the coxal fields I–II are sparsely punctate, while III–IV are densely punctate. In contrast, in females of *S. nucifragus*, the hypostomal apex is ornamented with two pairs of short protuberances; the length of setae *vi* is 150–200 µm; all coxal fields are apunctate. Furthermore, *S. euphonicus* sp. n. can be easily distinguished from other *Syringophilopsis* species parasitising fringillid birds, such as *S. kirgizorum* and *S. fringillae*, by the length of setae *f1* and *h1*. In females of *S. euphonicus* sp. n., setae *f1* and *h1* are long, exceeding 250 µm; in *S. kirgizorum*, setae *f1* and *h1* measure only 80 µm; in *S. fringillae*, setae *f1* are 185–190 µm long while setae *h1* measure 340–350 µm.

##### Etymology

The species name “*euphonicus*” is taken from the generic name of the hosts.

#### 3.1.3. *Syringophiloidus stawarczyki* Skoracki, 2004

This species was previously recorded on Euphoninae host, the Golden-rumped Euphonia *Chlorophonia cyanocephala* in Brazil [14], and two hosts from the family Thraupidae: the White-lined Tanager *Tachyphonus rufus* (Boddaert) and the Blue Dacnis *Dacnis cayana* (Linnaeus). Below, we present eight newly identified host species for this quill mite belonging to the *Chlorophonia* and *Euphonia* genera.

##### New Material Examined

Ex Blue-naped Chlorophonia *Chlorophonia cyanea* (Thunberg) (host uncatalogued); Brazil: Rio de Janeiro State, Rio de Janeiro, 20 July 1818, coll. J. Hatterer—fifteen females and one male deposited in the SNSB-ZSM (reg. no. ZSMA20250015), five females and one male in the AMU (reg. no. MS-24-1025-009). From the same host species (host reg. no. SNSB-ZSM 09/2278); Colombia: Bogotá D.C., Bogota, 1896, coll. C. Dalmas—six females deposited in the SNSB-ZSM (reg. no. ZSMA20250016), six females and one male in the AMU (reg. no. MS-24-1025-011).

Ex Velvet-fronted Euphonia *Euphonia concinna* (Sclater) (host reg. no. SNSB-ZSM 15.933); Colombia: Bogotá D.C., Bogota, no other data—ten females and one male deposited in the SNSB-ZSM (reg. no. ZSMA20250017), six females and one male in the AMU (reg. no. MS-24-1025-022).

Ex White-lored Euphonia *Euphonia chrysopasta* (Sclater & Salvin) (host reg. no. SNSB-ZSM 09.2363); Venezuela: Bolívar State, Río Caura, November 1897, coll. E. Andre—six females deposited in the SNSB-ZSM (reg. no. ZSMA20250018) and two females in the AMU (reg. no. MS-24-1025-042). From the same host species (host reg. no. SNSB-ZSM 27.1850); Bolivia: Santa Cruz Province, Buena Vista, August 1926, coll. M. Kiefer—ten females deposited in the SNSB-ZSM (reg. no. ZSMA20250019), seven females and one male in the AMU (reg. no. MS-24-1025-039). From the same host species (host reg. no. SNSB-ZSM 27.1849) and other data—two females deposited in the AMU (reg. no. MS-24-1025-040).

Ex Violaceous Euphonia *Euphonia violacea* (Linnaeus) (host reg. no. SNSB-ZSM 09.2373); Trinidad and Tobago: Trinidad Island, Santa Cruz, January 1897, coll. R. de Dalmas—four females deposited in the SNSB-ZSM (reg. no. ZSMA20250020) and two females in the AMU (reg. no. MS-24-1025-026).

Ex Thick-billed Euphonia *Euphonia laniirostris* (d’Orbigny & Lafresnaye) (host reg. no. SNSB-ZSM 09.2335); Venezuela: Sucre state, hills between Bermúdez and Cumaná, March 1897, coll. E. Andre—five females and one male deposited in the SNSB-ZSM (reg. no. ZSMA20250021), four females and two males in the (reg. no. MS-24-1025-031).

Ex Purple-throated Euphonia *Euphonia chlorotica* (Linnaeus) (host reg. no. SNSB-ZSM 32.1013); Paraguay: Apa Highlands (Apa Bergland) near Serranía San Luis National Park, Department of Concepción, 18 September 1931, coll. M. Kiefer—ten females and one male deposited in the SNSB-ZSM (reg. no. ZSMA20250022), four females and two males in the AMU (reg. no. MS-24-1025-020).

Ex Golden-rumped Euphonia *Chlorophonia cyanocephala* (Vieillot) (host reg. no. SNSB-ZSM 36.109); Paraguay: Dept. Itapúa, Cambyretá, 14 July 1936, coll. A. Neunteufel—ten females deposited in the SNSB-ZSM (reg. no. ZSMA20250023), eight females and one male in the AMU (reg. no. MS-24-1025-038). From the same host species (host reg. no. SNSB-ZSM 09/2316); Colombia: Bogotá D.C., Bogota, 1896, no other data—three females and one male deposited in the SNSB-ZSM (reg. no. ZSMA20250024), two females and one male (reg. no. MS-24-1025-036). From the same host species (host reg. no. SNSB-ZSM 09/2313) and other data—one female deposited in the AMU (reg. no. MS-24-1025-035).

#### 3.1.4. *Picobia chloris* Bochkov, Mironov & Kravtsova, 2000

This species was considered a monoxenous parasite, as it was previously known only from European Greenfinch *Chloris chloris* (Linnaeus) (Passeriformes: Fringillidae) in Kyrgyzstan [29]. Below, we report a new host species for this parasite.

##### New Material Examined

Ex Violaceous Euphonia *Euphonia violacea* (Linnaeus) (host reg. no. SNSB-ZSM 09.12); Brazil: Bahia State, no other data—three females and one male deposited in the SNSB-ZSM (reg. no. ZSMA20250025), two females (PF) and two males in the AMU (reg. no. MS-24-1025-030). From the same host species (host reg. no. SNSB-ZSM 09.2366); French Guiana: Cayenne, 1896, coll. Petit—one female deposited in the SNSB-ZSM (reg. no. ZSMA20250026) and one female in the AMU (reg. no. MS-24-1025-027). From the same host species (host reg. no. SNSB-ZSM 12.1682); Trinidad and Tobago: Trinidad Island, Caparo, 27 July 1912, coll. S. M. Klages—one female deposited in the AMU (reg. no. MS-24-1025-028).

### 3.2. Prevalence, Host Specificity and Bipartite Network Analysis

**Figure 6:**
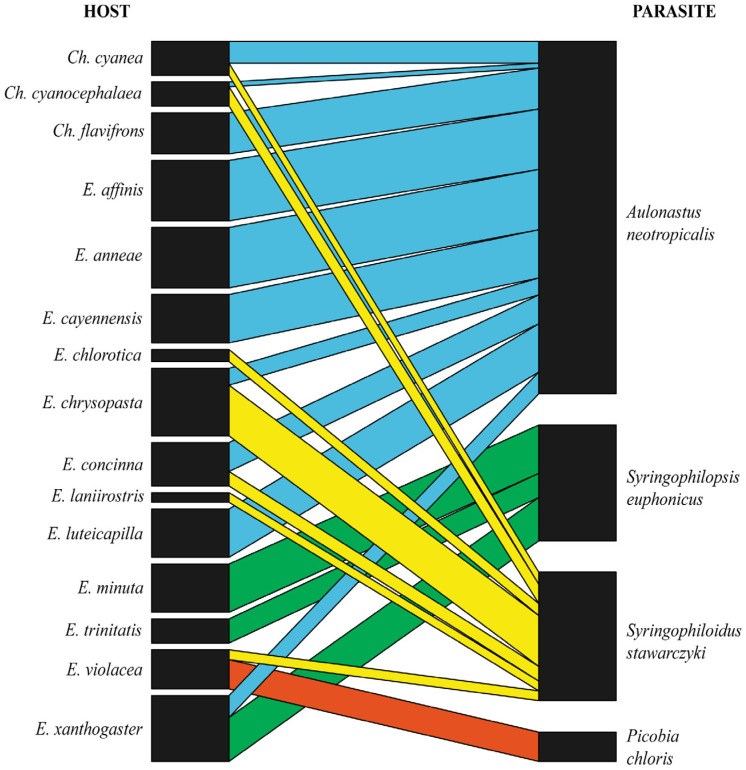
Bipartite network graph of interactions between Euphoninae hosts (left) and their quill mite species (right); edge thickness is correlated with prevalence.

Data from 298 euphonine individuals belonging to 25 species were included in this study. Out of these, 15 host species were found to be parasitised by 4 quill mite species from 4 genera belonging to the 2 subfamilies. Infestation prevalences ranged from 2% to 25% in particular host species (Table 1). Among the analysed material, 44 specimens belonging to 10 species were not infested by quill mites (Table 2). The syringophilid mites—Euphoninae bipartite network (Figure 6) exhibited a moderate value of connectance (Con = 0.35). It means that 35% of all possible connections between birds and quill mites are observed in the network. A value of C.score = 0.675 indicates a moderate tendency for non-co-occurrence, but a value of H2′ = 0.77 indicates moderate to high specialisation in the network. A comparison between H2′ and null model values revealed significant differences (mean H2′ for null model = 0.775; t = 0.006; *p* = 0.007), indicating a statistically significant deviation from randomness. Temperature of nestedness = 32.39 indicates moderate nestedness, i.e., the network is not perfectly nested but shows significant structure. The normalised specialisation level (d′) ranged from 0.62 to 0.91, indicating a network dominated by moderate to highly specialised interactions. This means that each of these parasite species generally has unique ways of interacting with the host in the network.

## 4. Discussion

The fauna associated with birds of the subfamily Euphoninae includes four species belonging to four genera and two subfamilies. Mites of the genera *Aulonastus* and *Syringophilopsis*, present on fringillid birds, are not exclusively associated with Euphoninae but have also been recorded on birds from the other two subfamilies, Fringillinae and Carduelinae [6,29,30,31,32,33]. The remaining two genera, *Syringophiloidus* and *Picobia*, are present on members of Euphoninae and Carduelinae but have not been found on representatives of Fringillinae [6,14,28,29,34,35]. Interestingly, despite a relatively large sample size (298 specimens belonging to 25 species), no representatives of the genera *Aulobia* and *Neopicobia*, which appear to be exclusively associated with members of Carduelinae [28,36,37,38,39], nor the genus *Torotrogla*, which inhabits birds belonging to both Fringillinae and Carduelinae [6,40,41], were found on Euphoninae (Table 3).

Molecular dating suggests that Euphoniinae diverged from Carduelinae approximately 13.8 million years ago, with the crown age of Euphoniinae estimated at 7.1 million years ago [42]. The subfamily Euphoninae includes only two genera, *Chlorophonia* and *Euphonia* [1,2], both of which originated relatively recently. The crown age of *Euphonia* is estimated at 6.5 million years, while *Chlorophonia* is estimated around 3.8 million years ago [42]. The recent analysis revealed that the ancestral lineage of Euphoninae likely migrated to the Neotropics from North America, eventually diversifying in South America after crossing the Isthmus of Panama. In the Neotropics, the formation of the Western Amazon basin and the Northern Andean uplift during the Miocene likely triggered vicariance events that drove the divergence of the *Chlorophonia* and *Euphonia* lineages. As a result, *Chlorophonia* adapted to the Andean region, while *Euphonia* established itself in the Amazon basin [3,42]. This biogeographical history likely shaped the distribution and diversity of their associated syringophilid mites.

Among quill mites, the species *Aulonastus neotropicalis* sp. n. and *Syringophiloidus stawarczyki* have been recorded on hosts from both genera and should be classified as mesostenoxenous parasites (more than one genus of hosts, but restricted to one family [39]). These species were likely present on the last common ancestor of the *Chlorophonia* + *Euphonia* lineage. In contrast, the species *Syringophilopsis euphonicus* sp. n. exclusively inhabits birds of the genus *Euphonia* and should be regarded as an oligoxenous parasite (more than one host, but restricted to one genus). Likely, this species was also present on the last common ancestor of the *Chlorophonia* + *Euphonia* lineage but during the divergence of the two genera, it failed to colonise *Chlorophonia* hosts. This could be attributed to the “missing the boat” phenomenon, in which a parasite present in an ancestral host fails to establish itself in one of the newly diverging lineages. Such failure may result from ecological constraints, host specialisation, or competition. In this case, *S. euphonicus* sp. n. may have been unable to compete with *Syringophiloidus stawarczyki*, which occupies the same habitat on Euphoninae hosts. The last recorded species of quill mites, *Picobia chloris*, has been found on only one host species of Euphoninae, i.e., *Euphonia violacea*. Interestingly, this species has also been recorded on *Chloris chloris*, a member of the subfamily Carduelinae [29]. In the case of *Euphonia violacea*, *P. chloris* was found on 3 individuals (out of 26 examined, prevalence = 12%) from three distinct localities: Brazil, French Guiana, and Trinidad and Tobago. It suggests that the mite forms a stable association with this host and should not be regarded as an incidental infestation. Unfortunately, the prevalence of *P. chloris* infestation in *Chloris chloris* remains unknown.

The analyses demonstrated that the quill mites—Euphonias network exhibits moderate specialisation, with connectance = 0.35. Within this system, all quill mite species, except *P. chloris*, interact with multiple hosts, yet there is still a notable degree of specialisation, as indicated by H2′ = 0.77 and d′ values ranging from 0.62 to 0.91. Generalists (in this case mesostenoxenous parasites), such as *Aulonastus neotropicalis* sp. n. and *Syringophiloidus stawarczyki*, infest a broad spectrum of birds belonging to both genera *Euphonia* and *Chlorophonia*. In contrast, specialists (oligoxenous parasites) are more selective, e.g., *Syringophilopsis euphonicus* sp. n., which is restricted to representatives of *Euphonia*. On the other hand, each host species has a unique parasite fauna, indicating that host–parasite interactions are not random but rather are shaped by evolutionary processes (e.g., co-speciation). Such a high degree of specialisation aligns with the structured interactions commonly observed in quill mites–birds relationships [9,10,11,12,13] and in other co-evolved warm-blooded vertebrates and obligatory parasitic mites [43,44,45,46,47,48]. The analyses also revealed that the C-score value of 0.675 is relatively high, suggesting that quill mites tend to avoid sharing the same hosts. Conversely, host species often harbour distinct parasite communities with minimal overlap. In this study, no single host species was infested by different quill mite species inhabiting the same niche. For instance, *Syringophilopsis euphonicus* sp. n. and *Syringophiloidus stawarczyki*, found in wing coverts, or *Aulonastus neotropicalis* sp. n. and *Picobia chloris*, inhabiting contour feathers, were never observed on the same host specimen or even host species. In cases where a host species was infested by multiple quill mite species, niche differentiation was always observed, with syringophilids occupying distinct ecological niches (types of feathers) on the same host to avoid competition. For example, *Chlorophonia cyanea*, *Ch. cyanocephala*, *Euphonia chrysopasta*, and *E. concinna* were infested by two different mite species occupying different habitats, with *A. neotropicalis* sp. n. always found in contour feathers and *S. stawarczyki* exclusively in wing coverts. It implies competitive exclusion among parasites, where quill mites avoid co-occurrence to minimise competition for resources on the same host. This strategy is frequently observed in host species infested by two, three, or even four syringophilid mite species [5,6].

## 5. Conclusions

In this study, we identified 4 species of quill mites parasitising 15 host species of Euphoninae, whereas previously, only a single quill mite species had been recorded from a single Euphoninae host. This significantly expands our understanding of syringophilid mite diversity in this bird group and highlights the complexity of their host–parasite associations. The phylogenetic and biogeographical history of Euphoninae has likely played a key role in shaping the distribution and specialisation of their associated parasites. While some species, such as *Aulonastus neotropicalis* sp. n. and *Syringophiloidus stawarczyki*, exhibit broad oligoxenous parasitism and were likely present on the last common ancestor of *Chlorophonia* and *Euphonia*, others—like *Syringophilopsis euphonicus* sp. n.—show a more restricted host association. The absence of *S. euphonicus* sp. n. in *Chlorophonia* may be attributed to the “missing the boat” phenomenon or competitive exclusion by *Syringophiloidus stawarczyki*. Network analysis of quill mite–Euphoninae interactions revealed a moderately specialised system, with most mites exploiting multiple hosts while maintaining distinct ecological preferences. The observed values suggest that quill mites tend to avoid sharing the same host, likely due to competitive exclusion and niche differentiation. The lack of co-occurrence among species occupying similar microhabitats, such as wing coverts or contour feathers, further supports this pattern. These findings align with broader host–parasite co-evolutionary trends, where specialisation and competition drive structured interactions within parasite communities.

## Figures and Tables

**Table 1:** Birds of the subfamily Euphoninae (Fringillidae) parasitised by quill mites of the family Syringophilidae with prevalence and distribution.

Host Species and No. of Examined Individuals	No of Infested	Prevalence and CI^(Sterne method)^	Mite Species	Locality
*Chlorophonia cyanea* (44)	4	9% (2.5–21.7)	*Aulonastus neotropicalis*	Bolivia, Venezuela
“	2	5% (0.6–15.5)	*Syringophiloidus stawarczyki*	Brazil, Colombia
*Chlorophonia cyanocephala* (40)	1	2% (0.1–13.2)	*Aulonastus neotropicalis*	Colombia
“	3	8% (1.6–20.4)	*Syringophiloidus stawarczyki*	Colombia, Paraguay
*Chlorophonia flavifrons* (6)	1	17% (0.4–64.1)	*Aulonastus neotropicalis*	Guadeloupe
*Euphonia affinis* (8)	2	25% (3.2–65.1)	*Aulonastus neotropicalis*	Colombia
*Euphonia concinna* (16)	2	12% (1.6–38.3)	*Aulonastus neotropicalis*	Colombia
“	1	6% (0.2–30.2)	*Syringophiloidus stawarczyki*	Colombia
*Euphonia luteicapilla* (5)	1	20% (0.5–71.6)	*Aulonastus neotropicalis*	Panama
*Euphonia anneae* (4)	1	25% (0.6–80.6)	*Aulonastus neotropicalis*	Costa Rica
*Euphonia xanthogaster* (22)	2	9% (1.1–27.6)	*Aulonastus neotropicalis + Syringophilopsis euphonicus*	Peru
“	2	9% (1.1–27.6)	*Syringophilopsis euphonicus*	Peru
*Euphonia chrysopasta* (14)	1	7% (0.2–33.9)	*Aulonastus neotropicalis*	Venezuela
“	3	21% (4.7–50.8)	*Syringophiloidus stawarczyki*	Bolivia, Venezuela
*Euphonia cayennensis* (5)	1	20% (0.5–71.6)	*Aulonastus neotropicalis*	French Guiana
*Euphonia trinitatis* (10)	1	10% (0.3–44.5)	*Syringophilopsis euphonicus*	Venezuela
*Euphonia minuta* (10)	2	20% (2.5–55.6)	*Syringophilopsis euphonicus*	Colombia
*Euphonia violacea* (26)	1	4% (0.1–19.6)	*Syringophiloidus stawarczyki*	Trinidad and Tobago
“	3	12% (2.4–30.2)	*Picobia chloris*	Brazil, French Guiana, Trinidad and Tobago
*Euphonia laniirostris* (25)	1	4% (0.1–20.4)	*Syringophiloidus stawarczyki*	Venezuela
*Euphonia chlorotica* (19)	1	5% (0.1–26.0)	*Syringophiloidus stawarczyki*	Paraguay

**Table 2:** Euphonine host species not infested by quill mites.

*Chlorophonia callophrys* (Cabanis), N = 8	*Euphonia gouldi* Sclater, N = 1
*Chlorophonia occipitalis* (Gisignies), N = 2	*Euphonia mesochrysa* Salvadori, N = 7
*Chlorophonia pyrrhophrys* (Sclater), N = 7	*Euphonia pectoralis* (Latham), N = 7
*Euphonia chalybea* (Mikan), N = 4	*Euphonia plumbea* Gisignies, N = 1
*Euphonia fulvicrissa* (Sclater), N = 3	*Euphonia rufiventris* (Vieillot), N = 4

**Table 3:** Quill mite genera associated with birds of the family Fringillidae; number of mite species/number of host species and distribution in the brackets.

Mite Genus	Euphoninae	Fringillinae	Carduelinae
*Aulobia*	-	-	2/7 (distribution: Holarctic)
*Aulonastus*	1/10 (Neotropical)	1/1 (Palaearctic)	1/2 (Palaearctic)
*Syringophiloidus*	1/8 (Neotropical)	-	4/5 (Palaearctic, Afrotropical)
*Syringophilopsis*	1/3 (Neotropical)	1/2 (Palaearctic)	1/5 (Palaearctic, Afrotropical)
*Torotrogla*	-	1/2 (Palaearctic)	3/9 (Holarctic)
*Neopicobia*	-	-	2/2 (Holarctic)
*Picobia*	1/1 (Neotropical)	-	1/1 (Palaearctic)

## Data Availability

All necessary data are available in the text.
